# The landscape of extrachromosomal circular DNA (eccDNA) in the normal hematopoiesis and leukemia evolution

**DOI:** 10.1038/s41420-022-01189-w

**Published:** 2022-09-28

**Authors:** Tiansheng Zeng, Wenhui Huang, Longzhen Cui, Pei Zhu, Qing Lin, Wenjuan Zhang, Junyi Li, Cong Deng, Zhihua Wu, Zeyong Huang, Zhiyong Zhang, Tingting Qian, Wei Xie, Min Xiao, Yingyu Chen, Lin Fu

**Affiliations:** 1 Department of Hematology, The Second Affiliated Hospital of Guangzhou Medical University, Guangzhou, Guangdong P. R. China; 2 Central Laboratory, The Second Affiliated Hospital, Guangzhou Medical University, Guangzhou, Guangdong P. R. China; 3 Translational Medicine Center, Guangzhou Key Laboratory for Research and Development of Nano-Biomedical Technology for Diagnosis and Therapy &Guangdong Provincial Education Department Key Laboratory of Nano-Immunoregulation Tumour Microenvironment, The Second Affiliated Hospital, Guangzhou Medical University, Guangzhou, Guangdong P. R. China; 4 Translational Medicine Center, Huaihe Hospital of Henan University, Kaifeng, Henan P. R. China; 5 Department of Hematology, Huaihe Hospital of Henan University, Kaifeng, Hena P. R. China; 6 Department of Clinical Laboratory, The Second Affiliated Hospital of Guangzhou Medical University, Guangzhou, Guangdong P. R. China; 7 Department of Hematology, Fujian Institute of Hematology, Fujian Medical University Union Hospital, Fuzhou, Fujian P. R. China

**Keywords:** Cancer genomics, Acute myeloid leukaemia

## Abstract

Elevated extrachromosomal circular DNA (eccDNA) has been reported to accelerate tumor pathogenesis. Although the eccDNA profiles of other tumors have been established, the landscape of the eccDNA of acute myeloid leukemia (AML) has not been revealed. Our study first depicted the eccDNA profile of normal hematopoiesis and AML evolution by exploiting the ATAC-seq and RNA-seq data from nine healthy donors and 12 AML patients, which contained a total of 137 cell samples and 96 RNA-seq samples (including 16 blood cell types of the normal hematopoietic and AML hierarchies). We found the number of eccDNAs generally increased with the evolution of normal hematopoiesis and AML. The ecDNAs and ring chromosomes were found to reappear both in normal hematopoiesis and AML cells. Furthermore, we compared the eccDNAs of AML with normal cells. There were almost 300 AML-specific genes, including the known oncogenes *NRAS*, *MCL1*, *EVI1*, *GATA2*, *WT1*, and *PAK1*. And the ecDNA (chr11: 58668376-58826008) occurred in five out of 17 AML evolution-related cells, which was associated with the high expression of the *GLYATL1* gene and the high expressed *GLYATL1* was a poor prognostic factor. In conclusion, the eccDNA profiles of normal hematopoiesis and AML evolution were depicted and the recurrent eccDNAs we revealed might be utilized in the treatment of AML as biomarkers.

## Introduction

Extrachromosomal circular DNA (eccDNA) was currently reported to generate in the process of DNA damage and the corresponding DNA repair [1, 2]. According to their different sizes and copy numbers, they can be divided into microDNA (<1 Mb) and ecDNA/ring chromosome (>1 Mb) [3–6]. The difference between ecDNA and ring chromosomes is ecDNA lacks centromeres and telomeres [7]. While the ring chromosome contains the centromeres and telomeres and is visible under the microscope [8]. Growing evidence identified that ecDNAs play a role in oncogenic functions, including oncogene amplification, tumor heterogeneity, oncogene transcription, drug resistance, and genomic rearrangement [9]. Several studies also verified oncogene amplification associated with eccDNA was rare in normal tissues but affluent in cancers. Nevertheless, ecDNA has been demonstrated to be associated with unfavorable prognosis in glioblastoma, sarcoma, esophageal carcinoma and so on [10]. Besides, the landscapes of eccDNA in neuroblastoma and glioblastoma were described [11, 12]. A previous study indicated that eccDNA amplification did not occur in blood or normal tissue [10]. Some other studies have confirmed that double minutes (DMs, a kind of ecDNA) in acute myeloid leukemia (AML) and myelodysplastic syndromes (MDS) are associated with micronuclei, *MYC* or *MLL* amplification, complex karyotype, monosomal karyotype, *TP53* deletion, and *TP53* mutations [13, 14]. Though there are some progress in the study of eccDNA in hematological malignancies, the landscape of eccDNA of AML and normal hematopoiesis have not yet to be fully clarified. Kumar et al. proved Assay for Transposase Accessible Chromatin with high-throughput sequencing (ATAC-seq) is a feasible and sensitive method to detect eccDNA in tumors, even for AML at the pre-amplification stage [15]. This provides us with a new perspective to explore the eccDNA profile in the evolution of AML and normal hematopoiesis.

This study analyzed the ATAC-seq data from all cells in normal hematopoietic and leukemia evolution to reveal the eccDNA landscape of normal hematopoiesis and AML.

## Results

### The recurrent eccDNA across all cell types of hematopoiesis evolution

**Fig. 1:**
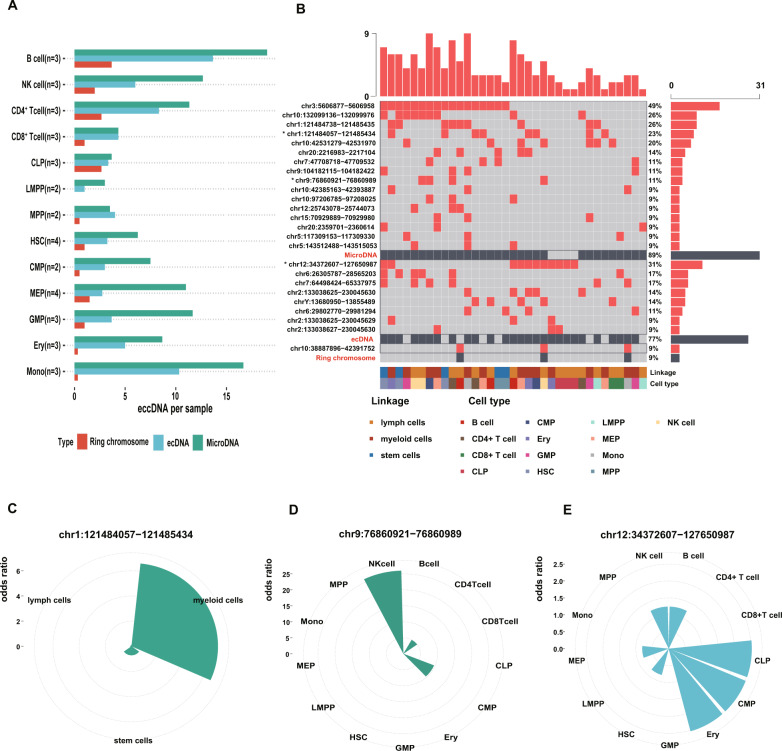
The recurrent eccDNAs across all cell types of normal hematopoiesis. **A** The barplot shows the average number of eccDNA in 13 cell types of hematopoiesis. The X-axis represents the cell type; Y-axis represents the average number of eccDNA (the eccDNA types are shown in different colors). **B** Oncoplot displays the eccDNA landscape of normal hematopoiesis. EccDNA on the left denotes “chromosome: chromosome start point−chromosome end point of breakage”, e.g., “chr3:5606877 − 5606958”. The eccDNAs are recurrent in different cell types and three type eccDNAs (microDNA/ecDNA/ring chromosome) were analyzed and ordered, respectively. The right bar represents the proportion of cells containing some kind of eccDNA in all normal cells. The samples at the bottom indicate according to the annotation bar (linkage and cell types). For example, the first line of the oncoplot shows the proportion of cells containing chr3:5606877 − 5606958 is 49%. **C** The barplot demonstrates microDNA (chr1:121484057 − 121485434) is enriched in myeloid cells (*P* = 0.039, Fisher’s exact test). **D** The microDNA (chr9:76860921 − 76860989) is enriched in NK cells (*P* = 0.025, Fisher’s exact test). **E** The ecDNA (chr12:34372607 − 127650987) is enriched in CLP (*P* = 0.020, Fisher’s exact test) and Ery cells (*P* = 0.020, Fisher’s exact test). In figure1C-E, the x-axis represents the different cells, the y-axis represents the odds ratio (OR).

The eccDNAs of 13 cell types of normal hematopoiesis evolution were shown in Fig. 1. On the whole, the average number of eccDNAs gradually increased as primitive cells differentiate into the terminal cells. Especially for microDNAs, the number of them was directly proportional to the degree of cell differentiation (Fig. 1A). The landscape of eccDNAs in normal hematopoiesis directly represented the recurrent eccDNAs. Three type eccDNAs (microDNAs/ecDNAs/ring chromosomes) were analyzed and ordered respectively. The Oncoplot showed the percentage of cells containing microDNA chr3:5606877−5606958 (or tandem gene duplication) in all cells was 49%, which indicated the microDNA chr3:5606877−5606958 was the most recurrent microDNA in normal hematopoiesis. All eccDNAs were ordered according to occurrence proportion (Fig. 1B). Subsequently, we analyzed all eccDNAs in Fig. 1B to explore which cell types were mainly enriched in. The microDNA chr1:121484057 − 121485434 (or tandem gene duplication) was enriched in myeloid cells (*P* = 0.039, Fig. 1C, Fisher’s exact test). The microDNA chr9:76860921 − 76860989 mainly occurred in the NK cells (*P* = 0.025, Fig. 1D, Fisher’s exact test). And the ecDNA chr12:34372607 − 127650987 tended to happen in CLP and Ery cells (both *P* < 0.05, Fig. 1E, Fisher’s exact test).

### The landscape of microDNAs across all cell types of normal hematopoiesis

**Fig. 2:**
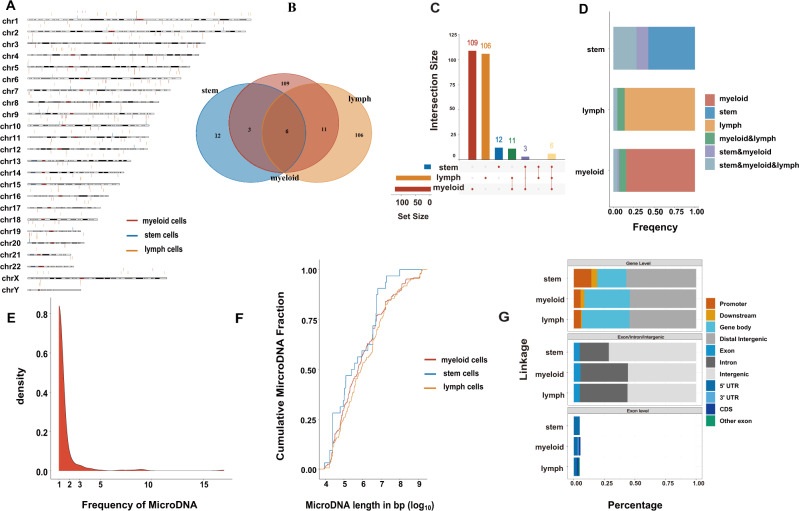
MicroDNA in the normal stem, myeloid, and lymphoid cells. **A** Chromosome ideogram plot showed the distribution of microDNA of stem, myeloid, and lymphoid cells. **B** Venn diagram of overlapping microDNA. **C** The barplot identified the microDNA number in the overlap of different cells. X-axis represents the cell type; Y-axis represents the average number of microDNA. **D** The frequency of overlapping microDNA in stem, myeloid, and lymphoid cells, respectively. **E** Frequency distribution graph showed that more than 80% microDNA only appeared once. **F** Length distribution of identified microDNA in stem, myeloid, and lymphoid cells. **G** microDNA distribution over different genomic features. At gene level, ~50% microDNA were mainly enriched in the promoter, downstream, and gene body. At exon/intron/intergenic level, most microDNA were enriched in intron and intergenic regions. At exon level, microDNA were mainly enriched in 5′ UTR.

To reveal the landscape of microDNAs in different normal blood cells, we proved the distribution of microDNAs of the stem, myeloid, and lymphoid cells by chromosome ideogram plot (Fig. 2A). By intersecting the microDNAs in the stem, myeloid, and lymphoid cells, we found that there were six overlapping microDNAs, which were far fewer than the special microDNAs in the myeloid and lymphoid cells, respectively. However, the overlapping part occupied a large proportion of microDNA in the stem cells. This indicated that a large part of the microDNA in the stem cells was recurrent in the lymphoid and myeloid cells, while most microDNAs in the lymphoid and myeloid cells were not similar to those in stem cells (Fig. 2B–D). Furthermore, the frequency distribution graph indicated that more than 80% microDNAs only occurred once (Fig. 2E). And the microDNAs of the stem cells had relatively long length than other two cell types’. Based on their genomic origin and genetic content, we studied the microDNA distribution over different genomic features. At gene level, ~50% microDNAs were enriched in the promoter, downstream, and gene body. And most microDNAs were enriched in intron and intergenic regions at exon/intron/intergenic level. At exon level, the microDNAs mainly distributed in 5′ UTR (Fig. 2F).

### The landscape of ecDNAs and ring chromosomes across all cell types in normal hematopoiesis

**Fig. 3:**
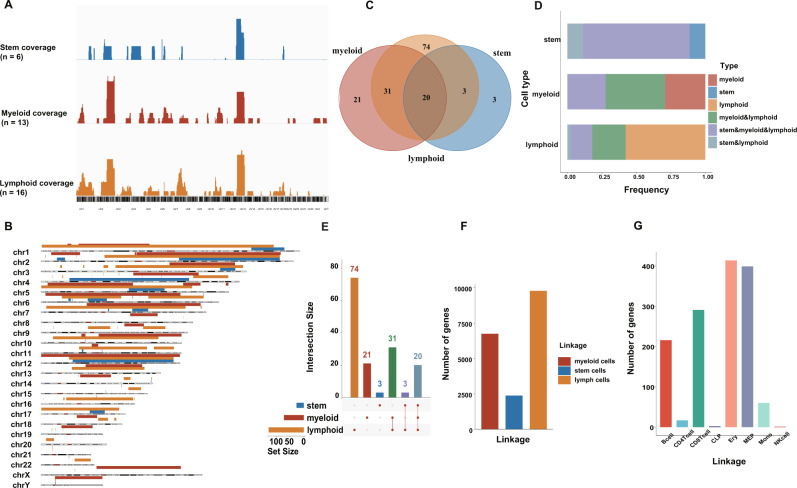
EcDNA and ring chromosome in the normal stem, myeloid, and lymphoid cells. **A** The frequency of ecDNA and ring chromosome on a different chromosome. **B** Chromosome ideogram plot presented the distribution of ecDNA and ring chromosome across the stem (*n* = 6), myeloid (*n* = 13), and lymphoid (*n* = 16) cells. **C** Venn diagram of overlapping ecDNA and ring chromosome. **D** The intersection size of overlapping ecDNA and ring chromosome in the stem, myeloid, and lymphoid cells, respectively. X-axis represents the cell type; Y-axis represents the intersection size. **E** The barplot identified the number of ecDNA and ring chromosomes in the overlap of different cells. X-axis represents the cell type; Y-axis represents the average number of ecDNA and ring chromosomes. **F** The average number of genes on ecDNA and ring chromosome in the stem, myeloid, and lymphoid cell, respectively. **G** The average number of genes on ecDNA and ring chromosome in B cell, CD4 + T cell, CD8 + T cell, CLP, Ery, MEP, Mono, and NK cell, respectively.

We also explored the landscape of ecDNAs and ring chromosomes and their functions. Of particular interest in these results was that the distribution frequency of ecDNA and ring chromosome was high in chromosome 2 and chromosome 12, especially in myeloid and lymphoid cells. Figure 3B illustrated the same result (Fig. 3A, B). Furthermore, the Venn diagram demonstrated overlapping ecDNAs and ring chromosomes of three cell types (The ratios of the overlapping ecDNAs and ring chromosomes in the stem cells, myeloid, and lymphoid cells were 76.9% (20/26), 27.8% (20/72), and 15.6% (20/128), respectively). The special ecDNAs and ring chromosomes in the stem cells, myeloid, and lymphoid cells, respectively accounted for 11.5% (3/26), 19.2% (21/72), and 57.8% (74/128). And Fig. 3D, E showed the same results (Fig. 3C–E). Besides, we also probe the average gene count of ecDNAs and ring chromosomes of the stem, myeloid, and lymphoid cells. The lymphoid cell samples had the largest number of genes, while the stem cell samples had the least count (Fig. 3F). This may be due to the stem cells having the least ecDNA and ring chromosome (Fig. 3E). To further investigate the genes distribution in different cells, we analyzed the average number of genes in B cell, CD4 + T cell, CD8 + T cell, CLP, Ery, MEP, Mono, and NK cell. There were more than 200 genes on ecDNA and ring chromosome of B cell, CD8 + T cell, Ery, and MEP (Fig. 3G).

### The recurrent eccDNAs across HSC, pHSC, LSC, and blast of AML evolution

**Fig. 4:**
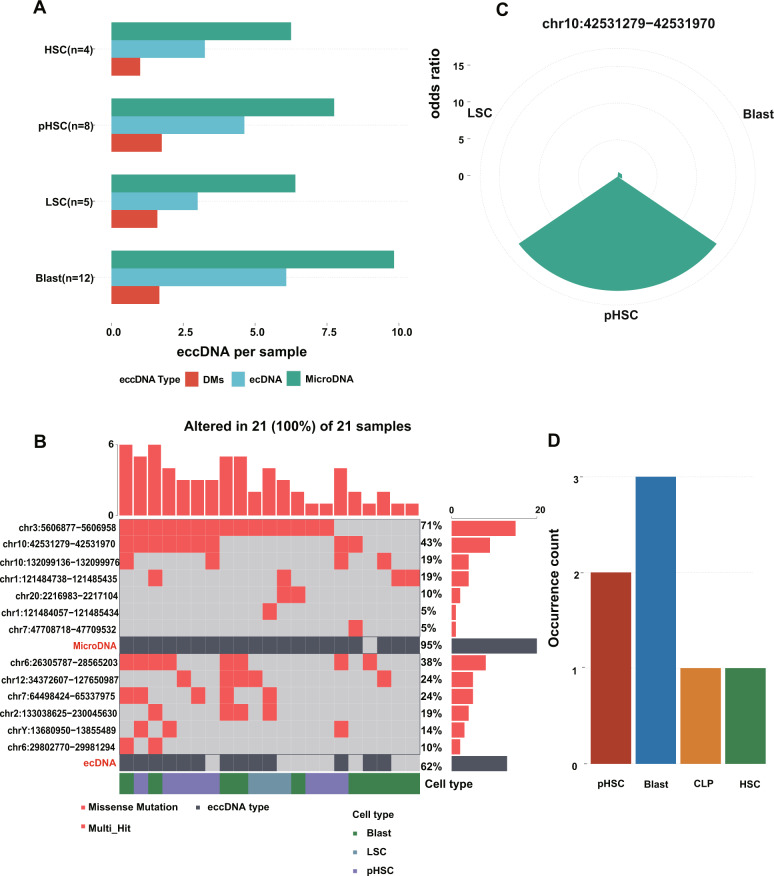
The recurrent eccDNA across four cell types of leukemia evolution. **A** The barplot revealed the average number of eccDNA in four cell types (including HSC, pHSC, LSC, and Blast) of leukemia evolution. The X-axis represents the cell type; Y-axis represents the average number of eccDNA (the eccDNA types are shown in different colors). **B** Oncoplot depicted the eccDNA landscape of leukemia evolution. The eccDNAs on the left were ordered according to the proportion of cells with that eccDNA in all cells. The samples at the bottom indicate according to the annotation bar (linkage and cell types). The sidebar plot represents the proportion of cells with eccDNA. **C** The barplot demonstrated micoDNA (chr10:42531279 − 42531970) was enriched in pHSC (*P* = 0.004). **D** The occurrence count of micoDNA (chr10:42531279 − 42531970) in all cells of normal hematopoiesis and leukemia evolution. The micoDNA (chr10:42531279 − 42531970) only occurred in HSC, CLP, pHSC, and blast. The X-axis represents the cell type; Y-axis represents the occurrence count of the micoDNA (chr10:42531279 − 42531970) in a different cell.

We continued to discover the landscape of eccDNAs in AML evolution. The average number of microDNAs (or tandem gene duplications) and ecDNAs gradually increased as primitive cells differentiate into the terminal cells except for LSC. And the number of ring chromosomes didn’t change much in four cell types (Fig. 4A). Oncoplot proved that cells with microDNA chr3:5606877 − 5606958 (or tandem gene duplication) accounted for 71%. All eccDNAs were ordered by proportion (Fig. 4B). Subsequently, all eccDNAs were used to conduct enrichment analysis. The microDNA chr10:42531279−42531970 (or tandem gene duplication) was enriched in pHSC (*P* = 0.004, Fig. 4C). We also analyzed the microDNA chr10:42531279 − 42531970 (or tandem gene duplications), which occurred in three blast cells, two pHSCs, one CLP and HSC (Fig. 4D).

### The landscape of microDNA across pHSC, LSC, and blast of AML evolution

**Fig. 5:**
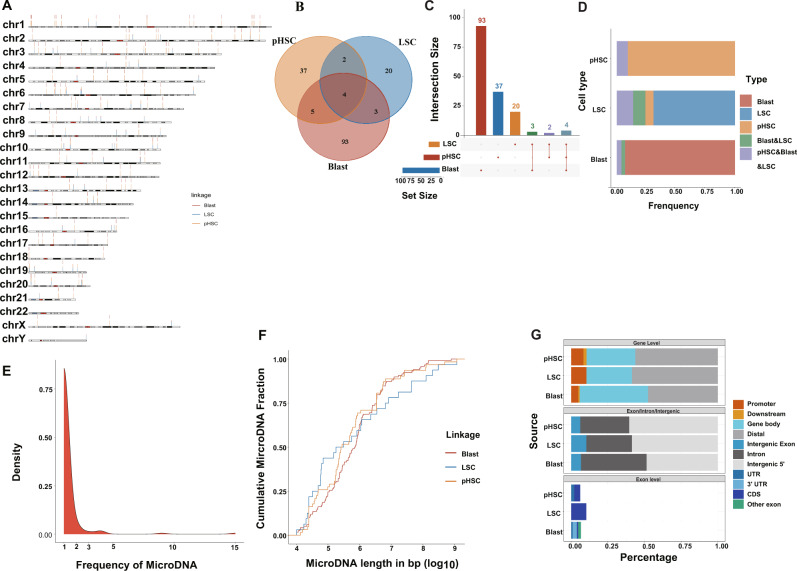
microDNA in pHSC, LSC, and blast. **A** Chromosome ideogram plot showed the microDNA distribution of pHSC, LSC, and blast. **B** Venn diagram of overlapping microDNA. **C** The barplot identified the microDNA number in the overlap of different cells. X-axis represents the cell type; Y-axis represents the number of microDNA. **D** The frequency of overlapping microDNA in pHSC, LSC, and blast, respectively. X-axis represents the cell type; Y-axis represents the intersection size. **E** Frequency distribution graph showed that more than 80% microDNA only appeared once in pHSC, LSC, and blast. **F** Length distribution of identified microDNA in pHSC, LSC, and blast. **G** Peak distribution over different genomic features. At gene level, ~50% microDNAs were mianly enriched in promoter, downstream, and gene body. At the exon/intron/intergenic level, most microDNAs were enriched in intron and intergenic regions. At exon level, microDNAs of pHSC were mainly enriched in 5′ UTR and CDS, microDNAs of LSC were mainly enriched in CDS. While microDNAs of blast were enriched in 3′ UTR and other exon.

We next dug the landscape of microDNA across HSC, pHSC, LSC, and blast in AML evolution. The distribution of microDNA across pHSC, LSC, and blast was shown in Fig. 5A. Furthermore, the Venn diagram and barplot confirmed that there were only four overlapping microDNA in pHSC, LSC, and blast, which demonstrated the high heterogeneity of microDNAs in three cell types (Fig. 5B, C). And the overlapping microDNA only accounted for a small part in all cell types (Fig. 5D). Then, we analyzed the occurrence frequency of microDNA in three cell types, and most microDNA tended to occur once (Fig. 5E). The length distribution of identified microDNA in pHSC, LSC, and the blast was showed in Fig. 5F. The microDNAs in pHSC and blast had a longer length. The peak distribution over different genomic features was also identified. At gene level, ~50% of microDNAs were mianly enriched in promoter, downstream, and gene body. At exon/intron/intergenic level, most microDNAs were enriched in intron and intergenic regions. At exon level, microDNAs of pHSC were mainly enriched in 5′ UTR and CDS, microDNAs of LSC were mainly enriched in CDS. While microDNAs of the blast were mainly enriched in 3′ UTR and other exons (Fig. 5G).

### The landscape of ecDNAs and ring chromosomes across pHSC, LSC, and blast of AML evolution

**Fig. 6:**
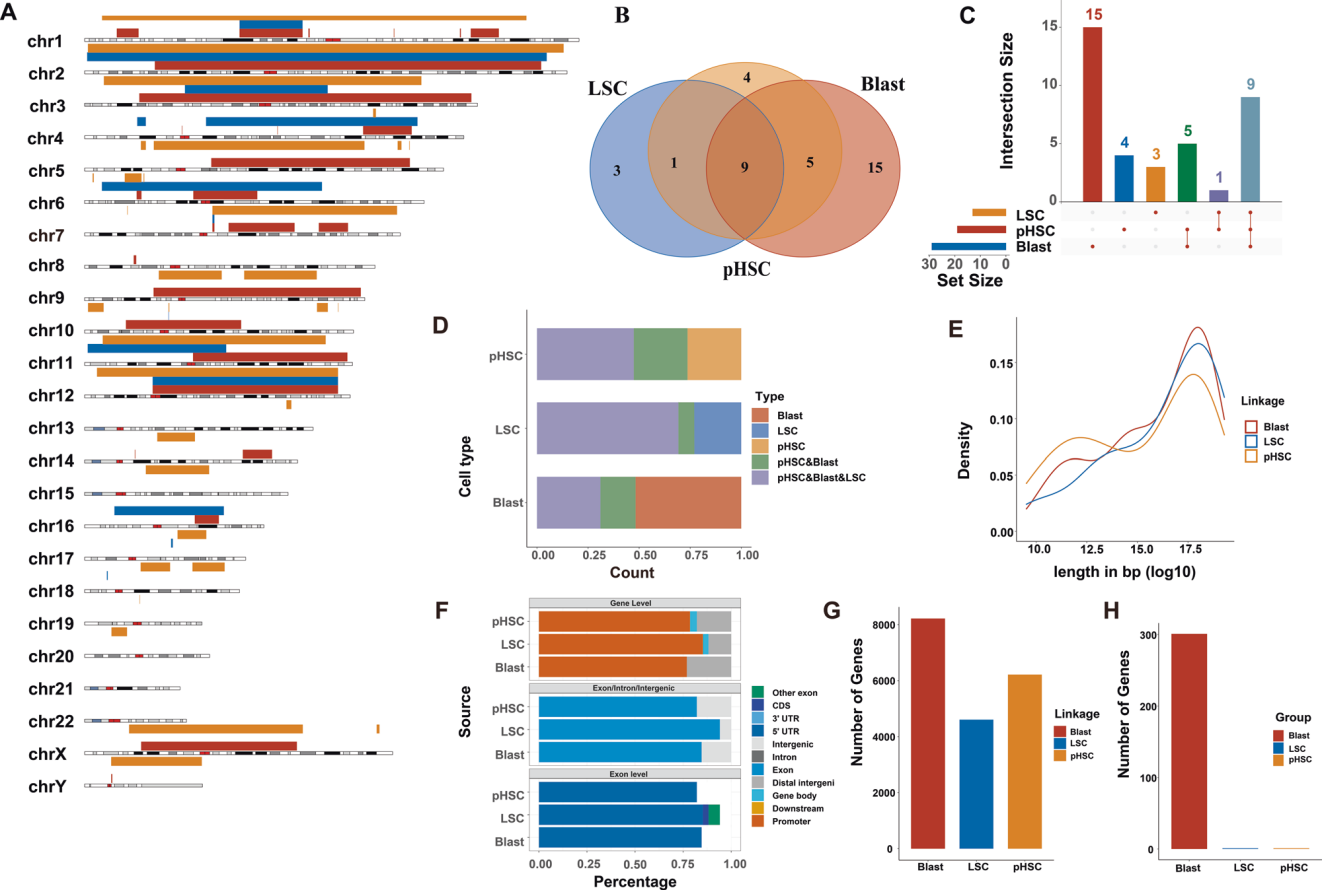
ecDNA and ring chromosome in pHSC, LSC, and blast. **A** The distribution of ecDNA and ring chromosome across pHSC, LSC, and blast. **B** Venn diagram of overlapping ecDNA and ring chromosome. **C** The barplot showed the ecDNA and ring chromosome number in the overlap of different cells. **D** The frequency of overlapping ecDNA and ring chromosome in pHSC, LSC, and blast, respectively. **E** The length distribution of ecDNA and ring chromosome in pHSC, LSC, and blast. **F** Peak distribution over different genomic features. **G** The average number of genes on ecDNA and ring chromosome in pHSC, LSC, and blast. **H** The number of blast-specific genes on eccDNA far more than pHSC and LSC.

The landscape of ecDNAs and ring chromosomes across pHSC, LSC, and blast of AML evolution was also investigated. The distribution of ecDNAs and ring chromosomes across pHSC, LSC, and blast was shown in Fig. 6A. Venn diagram and barplot manifested the number of overlapping ecDNAs and ring chromosomes in three cell types were nine, which occupied a large part of the ecDNAs and ring chromosomes of various cells, especially in LSC (Fig. 6B–D). Most ecDNAs and ring chromosomes were 10^17.5 bp in length (Fig. 6E). Moreover, we revealed the peak distribution over different genomic features. For all three cell types, at gene level, more than 75% of the ecDNAs and ring chromosomes were enriched in promoters. At exon/intron/intergenic level, ~80% of the ecDNAs and ring chromosomes were mainly enriched in exon. Then we analyzed the ecDNAs and ring chromosomes at the exon level and most of them were enriched in 5′ UTR (Fig. 6F). We further found that more than 8000 genes in blast cell, more than 4000 genes in LSC, and more than 8000 genes in pHSC (Fig. 6G). In Fig. 6H, there are ~300 blast-specific genes. The number of blast-specific genes was much higher than that in LSC and pHSC.

### The recurrent and specific eccDNAs in AML and normal cells

**Fig. 7:**
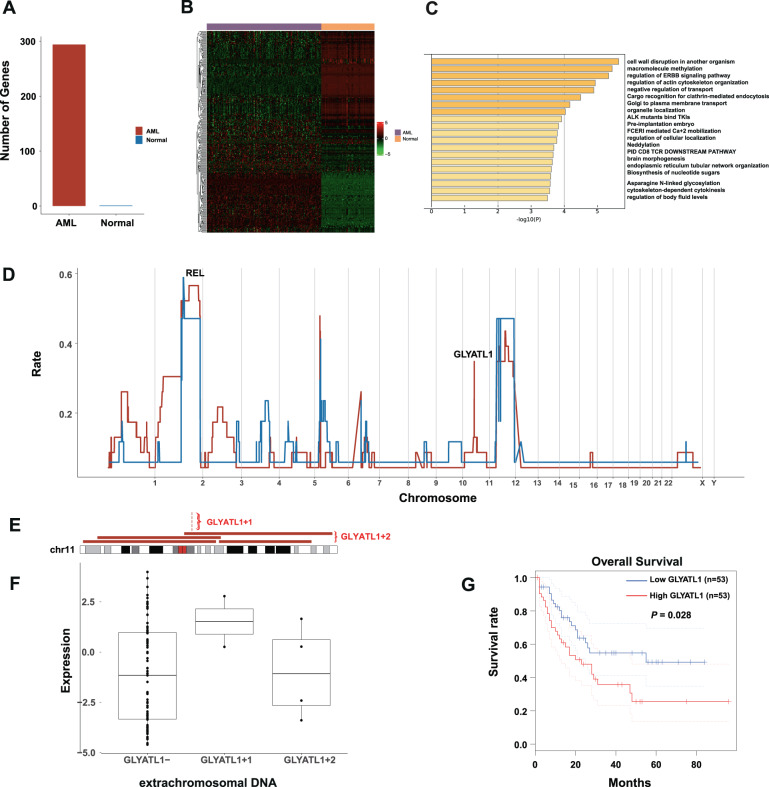
The comparison of eccDNA in AML cells (containing pHSC, LSC, and blast) and normal cells (including B cell, CD4 + T cell, CD8 + T cell, CLP, Ery, MEP, Mono, and NK cell). **A** The number of peculiar genes on eccDNA of AML cells is far more than normal cells. **B** Heatmap showed the cluster analysis of the different expressed AML-specific and normal-specific genes. The horizontal axis represents different genes, and the vertical axis represents AML or normal hematopoiesis. **C** GO enrichment of differential expressed genes on eccDNA of AML. **D** The rate of eccDNA in AML and normal cells. **E** Different lengths of eccDNA containing *GLYATL1*. *GLYATL1* + 1 represented shorter eccDNA that contain *GLYATL1*, *GLYATL1* + 2 represented longer eccDNA that contain GLYATL1. *GLYATL1*- represented eccDNA that doesn’t contain *GLYATL1*. **F** The *GLYATL1* + 1 expressed higher *GLYATL1*, and the expression of *GLYATL1* in the *GLYATL1* + 2 was almost the same as that of *GLYATL1*- (The *P* value was not significant). **G** The high expression of *GLYATL1* predicted shorter OS (*P* = 0.028).

To reveal the difference between eccDNAs in AML and normal cells, we found the number of genes on eccDNAs of AML was close to 300. The number was far more than normal cells (Fig. 7A). Heatmap showed the hierarchical clustering analysis of the different expressed AML-specific and normal-specific genes. The result demonstrated that AML-specific genes significantly differentiate the normal groups, and AML samples clustered tightly with each other. (Fig. 7B). Moreover, GO enrichment identified that differentially expressed genes on eccDNAs of AML were enriched in cell wall disruption in another organism, macromolecule methylation, regulation of ERBB signaling pathway, and regulation of actin cytoskeleton organization (Fig. 7C). The AML-related genes including *NRAS* (1p13.2), *MCL1* (1q21.2), *EVI1* (3q26.2), *GATA2* (3q21.3), *WT1* (11p13), and *PAK1* (11q14.1) were amplified in the eccDNAs of AML evolution compared with normal hematopoietic cells. The frequency of gene distribution of AML and normal cells displayed that REL was the most recurrent gene in eccDNAs of AML and normal cells. AML cells containing the eccDNA chr11: 58668376-58826008 highly expressed the *GLYATL1* gene, while the frequency of occurrence in normal cells was very low (Fig. 7D). Figure 7E depicted different lengths of eccDNA containing GLYATL1. We defined shorter eccDNA containing GLYATL1 as GLYATL1 + 1 and longer eccDNA containing GLYATL1 as GLYATL1 + 2. GLYATL1- represented eccDNA which doesn’t contain GLYATL1. Furthermore, GLYATL1 + 1 expressed the highest GLYATL1, while the expression of GLYATL1 on GLYATL1- and GLYATL1 + 2 was low (While the *P* value was not significant, Fig. 7F). Then, survival analysis demonstrated that AML patients with the higher expression of GLYATL1 had shorter OS (*P* = 0.028, Fig. 7G). We further analyzed the genes on eccDNA which were actually expressed and reported the pathways of those expressed genes. These actually expressed genes of eccDNA in normal hematopoietic cells and AML cells are shown in Supplementary Tables 2, 3, respectively. The GO enrichment showed the pathways of genes on eccDNAs of normal hematopoiesis and AML (Supplementary Figures).

## Discussion

We have gained a wealth of knowledge about tumor-related eccDNAs, especially the landscape of eccDNAs of glioblastoma [16]. However, there were few integral studies on the eccDNA profile of AML. Our study confirmed that eccDNA is indeed present in normal blood and AML. Furthermore, these eccDNAs might play an important role in AML evolution and normal hematopoiesis.

The previous study has proved that eccDNAs are common in normal hematopoietic cells [17]. In our study, there were recurrent eccDNAs in differently differentiated normal hematopoietic cells. The recurrent eccDNAs occupied a small percentage in myeloid and lymphoid cells, while accounting for a large proportion in stem cells. This might indicate that recurrent eccDNAs, especially recurrent microDNAs, decreased as the differentiation of blood stem cells and played an important role in this process. In addition, the recurrent ecDNAs and ring chromosomes were manifested to account for a large proportion of all eccDNAs in normal hematopoietic cells, which proved ecDNAs and ring chromosomes might have played a greater role in the evolution than microDNAs. Then our study indicated the more differentiated cells had a greater amount of eccDNA, especially the microDNAs. And microDNAs of normal hematopoiesis were mainly enriched in the 5’ UTR, which was consistent with previous findings [6]. However, we found the recurrent microDNAs of pHSC, LSC and blast were very rare and more than 75% of the microDNAs only occurred once. We tended to believe that the microDNAs were not the driver eccDNAs to promote the progression of AML. Our results proved the eccDNAs were common in normal hematopoietic cells and they were essential in normal hematopoiesis.

In AML evolution, the eccDNAs also increased with the evolution from HSC to blast, except for LSC. A recent study revealed that AML patients with double minutes (DMs, a kind of ecDNA) presented an extremely poor prognosis [14]. Approximately 30% of the ecDNA were paired with DMs [18]. Therefore, the increased ecDNAs, such as DMs might accelerate the progression of AML. We also disclosed more than 75% of the ecDNAs and ring chromosomes were enriched in promoter, exon, and 5′UTR. There was a study indicating the promoter eccDNAs can be re-inserted into other types of eccDNAs to generate larger eccDNAs called function-enhanced eccDNAs. These factors could be served as the genetic basis for the functional and numerical diversity of eccDNAs, and contribute to their structural diversity [19]. GO enrichment displayed that AML-specific genes were mainly enriched in keratinization, which is associated with a poor prognosis in lung squamous cell carcinoma [20]. We further compared the eccDNAs of AML with normal hematopoietic cells. Of particular interest in this context is the number of AML-specific genes is far more than the normal cell-specific genes. Among them, AML-specific genes *NRAS*, *MCL1*, *EVI1*, *GATA2*, *WT1*, and *PAK1* could promote the development and invasion of AML [21–26]. Besides, we also found that glycine-*N*-acyltransferase like 1 (*GLYATL1*), occurred in five AML evolution-related cells. *GLYATL1* only highly expressed in AML cells and AML patients with the high expression of *GLYATL1* had a shorter OS. *GLYATL1* was also reported to overexpress in primary prostate cancer [27]. The previous study showed that eccDNA amplification frequently occurred in many cancer types but wasn’t reported in hematological malignancies [10]. Therefore, we speculated that these genes in eccDNA of AML might accelerate the AML progression through the effects of poor prognostic factors, including complex karyotypes, monosomal karyotypes, *TP53* deletion, and *TP53* mutations. These studies also reported DMs in myeloid neoplasms commonly harbored *MYC*, *KMT2A*, or *MLL* gene amplification [13, 14]. Whether the oncogene amplifications of eccDNAs occurred in myeloid tumors still needs further exploration.

In conclusion, the eccDNAs generally increased with the evolution of normal hematopoiesis and AML. There were some recurrent eccDNAs both in normal hematopoiesis and AML cells, especially ecDNAs and ring chromosomes. Whether it’s the intra-group comparison in AML cells or the comparison between AML and normal hematopoietic groups, we found that AML blast-specific genes and AML-specific genes were much more than in other groups. Combined with the previous studies, the accumulation of eccDNAs and the oncogenes (*NRAS*, *MCL1*, *EVI1*, *GATA2*, *WT1*, *PAK*1, and *GLYATL1*) in the eccDNAs of AML evolution might contribute to AML progression. Moreover, we speculated that the high expression of AML-specific oncogenes in eccDNA might be associated with some inferior prognostic effects to promote AML progression. *GLYATL1* might be a prognostic biomarker in AML. However, our algorithm cannot distinguish between extrachromosomal circles and chromosomal segmental tandem duplications unless the circles are experimentally purified prior to library preparation to remove the linear DNA, and most tandem duplications tend to be short segments. So we refer to these eccDNA as microDNA or tandem duplications. It’s necessary to provide more evidence and information for further research.

## Method

### Patients and samples

In this study, ATAC-seq data from cells isolated from 9 healthy human donors (Donor5852, Donor6792, Donor7256, Donor7653, Donor1022, Donor4983, Donor2596, Donor5483, and Donor6926) and 12 patients with AML (SU654, SU353, SU444, SU209, SU575, SU070, SU351, SU583, SU501, SU484, SU496, and SU048). A total of 137 cell samples contained 16 blood cell types of the normal hematopoietic and AML hierarchies. Thirteen cell types were normal hematopoietic cells, including hematopoietic stem cell (HSC), multipotent progenitor (MPP), lymphoid-primed multipotent progenitor (LMPP), common myeloid progenitor (CMP), granulocyte-macrophage progenitor (GMP), megakaryocyte-erythroid progenitor (MEP), monocyte (Mono), erythroid (Ery), common lymphoid progenitor (CLP), CD4^+^ T cell (CD4), CD8^+^ T cell (CD8), B cell (B), and natural killer (NK) cell. The LMPP, CD4, CD8, B, and NK cells belong to lymphoid cells. The CMP, GMP, MEP, Mono, and Ery cells are part of myeloid cells. The remaining three cell types of AML evolution are preleukemic HSC (pHSC), leukemia stem cell (LSC), and leukemia blast cell (blast). These samples were exploited to ATAC-seq. And the paired expression data of 96 samples from RNA-seq was enrolled in our study. All original ATAC-seq and RNA-seq data were available under GEO accession GSE74912. Above AML samples were from the article of Corces et al. and obtained from patients at Stanford Medical Center with informed consent and agreement from institutional review board (IRB)-approved protocols (Stanford IRB, 18329 and 6453) [28]. Other 106 AML patients for survival analysis from The Cancer Genome Atlas (TCGA) database (https://cancergenome.nih.gov).

### Fast-ATAC sequencing

All cell samples were separated by flow cytometry analysis and cell sorting (FACS). ATAC-seq data in our study was derived from the fast-ATAC sequencing, which is an optimized protocol for blood cells and requires just 5000 cells. Five thousand cells were pelleted by centrifugation at 500×*g* RCF for 5 min at 4 °C and removed all supernatant. Then added 50 ul transposase mixture (25 μl of 2× TD buffer, 2.5 μl of TDE1, 0.5 μl of 1% digitonin, and 22 μl of nuclease-free water) (FC-121-1030, Illumina; G9441, Promega) in the cells and mixed well. Transposition reactions were incubated at 37 °C for 30 min with agitation at 300 rpm, afterwards purified DNA and prepared the library. More detailed steps of the protocol can be found in this article [28]. ATAC-seq data was analyzed by the previous method [29], with the only exception is that reads were trimmed using a custom script and aligned using Bowtie2.

### Detection of the eccDNA circles from ATAC-seq data

All ATAC-seq data were processed as previously described [15]. Using bwa-mem [30], with the default setting to map paired-ended reads to the hg19 genome build. The split reads were collected using the tool samblaster [31]. The complete pipeline to identify the eccDNAs coming from one locus of any length is available through the GitHub (https://github.com/pk7zuva/Circle_finder and https://github.com/pk7zuva/Circle_finder/blob/master/circle_finder-pipeline-bwa-mem-samblaster.sh). Obtained the eccDNAs were annotated by annovar with refGene and cytoBand (https://annovar.openbioinformatics.org/en/latest/). Oncogene and tumor suppression genes were annotated according to oncokb (https://www.oncokb.org/).

### Characterize eccDNA across all cell types

The eccDNAs were dived into mircroDNAs and ecDNAs according to length. Most statistical analyses in this study were performed and visualized by the R Bioconductor package, Maftools package [32]. Oncoplot of the eccDNA across all cell types identified by Maftools visualization. Karoplot of eccDNA was performed by karyoploteR. The various groupwise and pairwise comparisons were performed to identify enriched eccDNA for every category cell. Overlapping eccDNA regions across cell types and the eccDNA distribution over different genomic features were analyzed by ChIPpeakAnno. GO enrichment was processed by Metascape (http://metascape.org/).

### Survival analysis

The survival analysis was performed by Kaplan–Meier method in GEPIA (http://gepia.cancer-pku.cn/), the data from The Cancer Genome Atlas (https://portal.gdc.cancer.gov/). The endpoint was overall survival (OS). OS was defined as the time from study enrollment to death or last follow-up. The *P* value with statistical significance was 0.05 for the two-tailed test and the confidence interval (CI) was 95%.

## Supplementary information

Supplementary figures

Supplementary table 1

Supplementary table 2

Supplementary table 3

## Data Availability

All original ATAC-seq and RNA-seq data were available under GEO accession GSE74912.
