# Supplementary material for: The landscape of extrachromosomal circular DNA (eccDNA) in the normal hematopoiesis and leukemia evolution
Source: Cell Death Discov. 2022 Sep 28;8:400. doi: 10.1038/s41420-022-01189-w (PMC9519993; doi:10.1038/s41420-022-01189-w)
Supplement: Supplementary file 1 — Supplementary figures [file 41420_2022_1189_MOESM1_ESM.pdf]

Supplementary figures

A

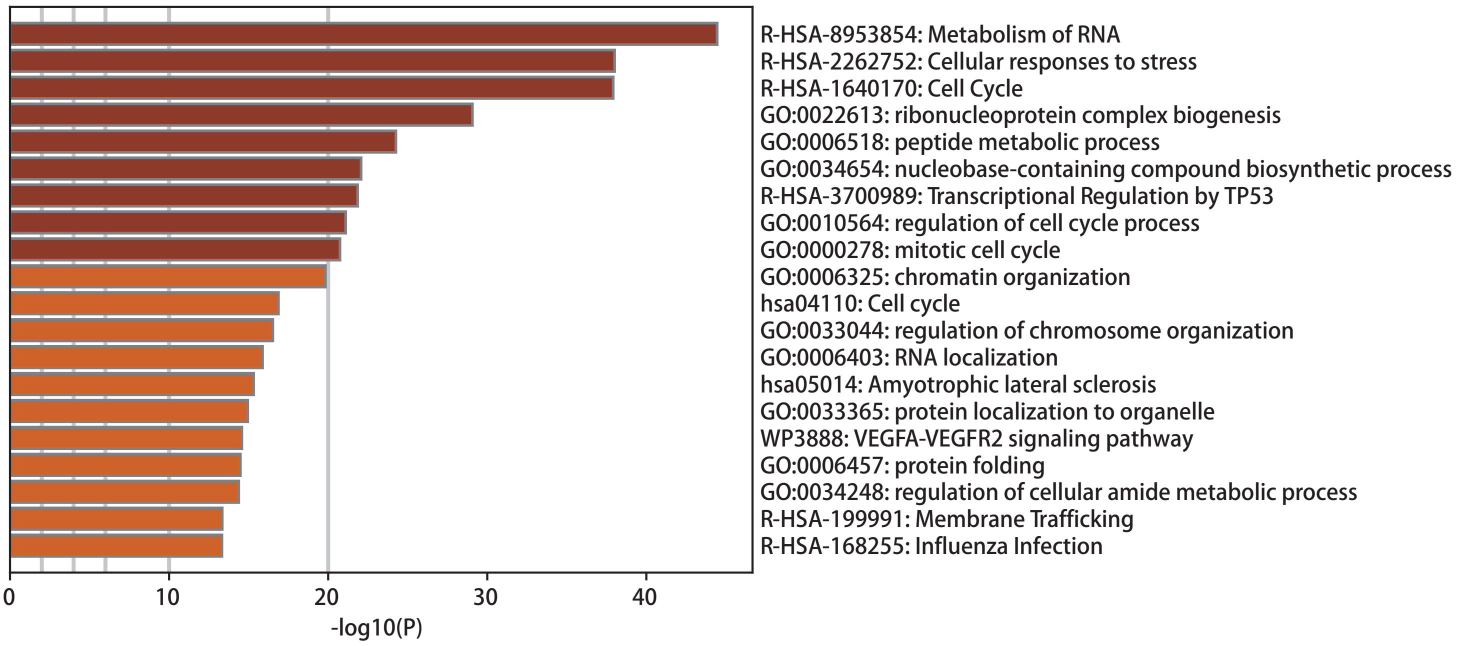

B

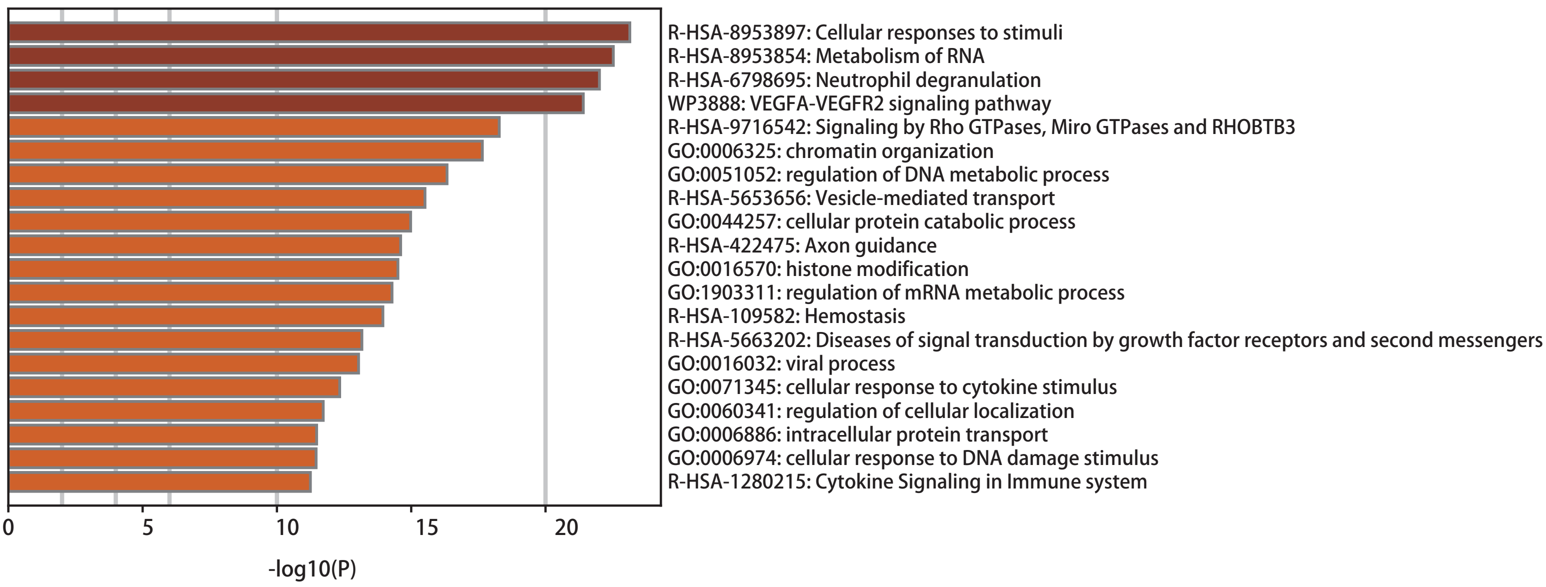

A. GO enrichment of actually expressed genes of eccDNA of normal hematopoietic cells
B. GO enrichment of actually expressed genes of eccDNA of AML cells
